# Predicting timely transfer to adult care in a cohort of autistic adolescents and young adults

**DOI:** 10.1371/journal.pone.0289982

**Published:** 2023-09-13

**Authors:** Laura C. Hart, Joseph Sirrianni, Steve Rust, Christopher Hanks

**Affiliations:** 1 Nationwide Children’s Hospital, Columbus, OH, United States of America; 2 Department of Internal Medicine, The Ohio State University College of Medicine, Columbus, OH, United States of America; University of South Carolina, UNITED STATES

## Abstract

**Background:**

The transition from pediatric to adult care is a challenge for autistic adolescents and young adults. Data on patient features associated with timely transfer between pediatric and adult health care are limited. Our objective was to describe the patient features associated with timely transfer to adult health care (defined as </ = 6 months between first adult visit and most recent prior pediatric visit) among a cohort of autistic adolescents and young adults.

**Methods and findings:**

We analyzed pediatric and adult electronic medical record data from a cohort of adolescents and young adults who established with a primary-care based program for autistic adolescents and young adults after they transferred from a single children’s hospital. Using forward feature selection and logistic regression, we selected an optimal subset of patient characteristics or features via five repetitions of five-fold cross validation over varying time-frames prior to the first adult visit to identify patient features associated with a timely transfer to adult health care. A total of 224 autistic adolescents and young adults were included. Across all models, total outpatient encounters and total encounters, which are very correlated (r = 0.997), were selected as the first variable in 91.2% the models. These variables predicted timely transfer well, with an area under the receiver-operator curve ranging from 0.81 to 0.88.

**Conclusions:**

Total outpatient encounters and total encounters in pediatric care showed good ability to predict timely transfer to adult health care in a population of autistic adolescents and young adults.

## Introduction

Approximately 50,000 autistic people reach adulthood each year, [1] and with adulthood comes the transition to adult health care. This transition is especially challenging for autistic adolescents and young adults, who must navigate loss of school support services and changes in insurance at the same time that they must find a new medical team in the adult world [2]. Legal privacy protections that occur when an adolescent reaches age 18 also create challenges in the form of communication barriers between the medical team and an adolescent’s parents, who have frequently been routinely involved in their child’s care up until that age [3]. As a result of these and other barriers, autistic adults experience poor health outcomes during transition, including unmet medical needs, [4] missing preventive services, [4] and more frequent visits to the emergency department [5–7].

A number of studies have evaluated the barriers to successful transition and transfer and identified individual-, provider-, and systems-level barriers to care [4, 8–10]. Less is known about the factors associated with successful transition and transfer to adult health care. In particular, guidelines and expert statements recommend that patients go no more than 3–6 months between their last pediatric and first adult visit (known as having a timely transfer), [11–13] but patient-level factors associated with a transfer occurring in this time frame are not well-described. In this study, we sought to evaluate the patient-level factors associated with the timely transfer from pediatric to adult health care for a cohort of autistic adolescents and young adults.

## Methods

### Patient population

To be included in this study, patients had to have at least one encounter at Nationwide Children’s Hospital (NCH) prior to at least one encounter at The Center for Autism Services and Transition (CAST), based at The Ohio State University Wexner Medical Center in Columbus, Ohio. CAST is a primary-care based program that was established to provide primary care to autistic adults in a way that is adapted to the unique needs of this population and with particular attention to their transition to adult care [9, 14]. Due to the geographic proximity of NCH and CAST (less than 15 miles), many CAST patients have been referred to CAST by an NCH clinician. Thus, this population is well-suited for studying the transfer to adult health care.

### Ethics considerations

This study was approved by the Nationwide Children’s Hospital Institutional Review Board with a waiver of consent due to the retrospective nature and minimal risk of the research.

### Dataset formation

To acquire the dataset, the patients in CAST were cross-referenced with the historic patients at NCH, matching by name, date-of-birth, and other identifying information by an honest broker who then provided a deidentified data set to the study team. For all matched patients, data from the electronic medical record (EMR) from both institutions for the dates January 2011 to May 2020 were pulled and linked so that analyses could be performed across both institutions over time. Analyses were completed using this linked data set.

### Feature extraction

We developed several categories of features to serve as candidate predictors of a timely transfer. These categories are: encounter features, vaccine features, patient vitals, completion of blood work (yes/no), insurance type (e.g. Medicare, Private, Uninsured, etc.), messages in the patient portal, prescribed medications, and International Classification of Diseases, Tenth Revision (ICD-10) billing codes. Specific features within these categories are:

- Encounter features include counts of patient encounters of different types. We derived four features to count the number of in-patient encounters, out-patient encounters, emergency encounters and a total count of all encounters for a patient.
- We incorporated vaccines features in multiple ways. We assessed vaccines individually if they were administered during the study period. We also created two binary features representing a patient’s vaccination status. The features are a “Minimal Vaccine Requirement,” which consists of having a record of a Tetanus/ diptheria / acellular pertussis (also called Tdap) and Meningococcal vaccine, and “Normal Vaccine requirement,” which consists of the minimum requirement plus at least one Human Papilloma Virus (HPV) vaccination. Given inconsistent incorporation of historical vaccines into the EMR, we felt that using a more comprehensive assessment of vaccine status would be biased due to missing data.
- Patient vitals consisted of nine total features: a patient’s body mass index (BMI) at or prior to their last pediatric visit, average blood pressure (systolic), average blood pressure (diastolic), the standard deviation of their systolic blood pressure, the standard deviation of their diastolic blood pressure, average pulse, the standard deviation of their average pulse, average temperature, and a binary feature for if their blood pressure was ever taken. For the average and standard deviation features, the values were calculated across all measurements from patient encounters in the time frame.
- The completion of bloodwork features are two binary features indicating if a patient 1) had bloodwork done, and 2) had bloodwork done in an outpatient encounter (outside of a hospital encounter).
- The insurance type features consisted of seven features: the total number of unique insurance types a patient had on record as using, and binary features (1 if used, 0 if not) for each insurance type a patient used on record (Medicare, Medicaid, Private Insurance, Other government insurance, Uninsured, or Other).
- The messages in patient portal feature was a binary feature classifying patients based on whether they had ever received a message in their patient portal.
- To allow for the incorporation of medications in a clinically meaningful way and to collapse the number of medication features included, medications were classified by 2 of the authors (L.H and C.H.) into their drug class, such as “antibiotic” or “anti-epileptic.” These were further collapsed into 32 higher-level groupings. For example, the “non-steroidal anti-inflammatory drug (NSAID)” drug class and the “opioid” drug class were collapsed into the higher-level grouping of “pain medications.” We opted for this strategy rather than using standard medication taxonomies as some common medications are used for uncommon indications in autistic people. For example, naltrexone, an opioid antagonist primarily used for the treatment of opioid use disorder and alcohol use disorder is used as a treatment for behavior problems in some autistic people [15, 16].
- We created the ICD-10 Billing Code features to represent the types of diagnoses associated with each patient. Due to the large number of ICD-10 codes present in the dataset, we needed to collapse the ICD-10 codes into groups. To do this, we mapped each ICD-10 diagnosis code to its corresponding Category in the ICD-10 hierarchy using the 2022 ICD-10 tables published by the Centers for Medicare & Medicaid Services (CMS). All historic ICD-9 codes were mapped to their appropriate ICD-10 code using the CMS 2018 General Equivalence Mappings. In total there were 32 billing code features, each coded in a binary way (i.e. the patient did or did not have that billing code feature in their medical record).

Each patient’s features were extracted by looking at their NCH data up to six years prior to their first CAST encounter. As a sensitivity analysis, we varied the time-window of NCH data that was used from 2 to 6 years during model training. Adjusting the time-window increases both the total number of varying features because medications and billing codes are added into the feature set as the time window is extended and the total number of patients because patients that only have NCH data from further back in time will only appear in longer time window analyses. The trade-off between shorter and longer time windows is that longer windows have more data points, but they might be less relevant the further back in time they occur. Thus, we chose to identify predictive features across multiple time window sizes to best account for these trade-offs. Table 1 breaks down the number of patients and number of features for each time window considered. Each feature was normalized using min-max normalization to be within the range 0–1, which typically helps model training and improves model performance [17].

**Table 1:** Breakdown of the number of patients and the size of their feature sets for each dataset based on their time window.

Years Prior to First CAST visit Considered	Patients			Number of Varying Features
	Total	# with a Timely Transfer (%)^a^	# without a Timely Transfer (%)^b^	
2	186	126 (67.7%)	60 (32.3%)	196
3	201	126 (62.7%)	75 (37.3%)	210
4	212	126 (59.4%)	86 (40.6%)	217
5	220	126 (57.3%)	94 (42.7%)	221
6	224	126 (56.3%)	98 (43.8%)	221

a. Defined as </ = 6 months between last pediatric visit and first CAST visit

b. Defined as > 6 months between last pediatric visit and first CAST visit

### Model development and training

We trained logistic regression models using forward variable selection to predict if a patient would have a timely transfer from pediatric care to the CAST program based on their historic NCH data. We defined a timely transfer as the patient having their first CAST encounter within six months of their most recent NCH encounter.

Our original plan was to train a regularized logistic regression model on our entire feature set (described above). However, when we attempted to train such a model we found that, due to the small number of patients relative to the large number of features (196 features), the model fitting algorithms failed to converge because of qusi-separability [17]. As a result, we opted for a different approach.

To counteract the quasi-separability and deal with a large amount of correlation between the features (which can cause other convergence issues), we adjusted our modeling methodology to include forward feature selection [18]. We started with an empty model, then added features, one at a time, according to which feature increased the Akaike Information Criterion (AIC) the most, until no remaining candidate features would increase AIC further. For each model along the forward selection process (one model per added feature), we calculated the area under the receiver-operator [ROC] curve (AUC) based on the combined hold-out data from a 5-fold cross validation. Finally, we selected the model with the best AUC as our final model. This techniques allows us to only keep features that are the most relevant for the prediction problem, while avoiding convergence errors caused by quasi-separation and correlations between features. For each time window (2–6 years prior to the first CAST visit), we trained models using five repetitions of five-fold cross validation to ensure the prediction performance and selected feature sets were stable across different subsets of the data. This resulted in 125 models (5 time windows * 5-fold cross-validation * 5 repetitions).

## Results

Table 2 shows the demographic characteristics for the cohort, which are notable for being mostly white (70%) and mostly male (84%).

**Table 2:** Demographic features of the cohort (n = 224).

Mean age at last pediatric visit (IQR)	18.6 years (2.9 years)
Male gender (%)	189 (84.4)
Race (%)	
Black	35 (15.6)
White	157 (70.1)
Insurance ^a^	
Public (Medicare/Medicaid) (%)	167 (74.6)
Private (%)	148 (66.1)
Patients with a Timely Transfer (%)	126 (56.3)
Median time to Transfer (IQR)	136 days (434 days)

a. Note that percentages add up to > 100% due to patients having multiple kinds of insurance.

Table 3 presents the features selected most often across all models in the first 5 steps of the forward selection. The feature “Number of Outpatient Encounters” was selected in 68% of the models at Step 1, and “Number of Total Encounters” was selected in 23.2% of the models at Step 1. These two variables are highly correlated in this data set (r = 0.99), and so together were the feature selected at Step 1 in over 90% of the models and were selected in over 96% of the models by Step 5. No other features were selected this frequently by Step 5 of the model, and none of the other features selected most frequently correlated as highly with each other.

**Table 3:** Features selected most often in the first five steps of the forward selection across all models.

Feature	Percentage of Models the feature was selected on or before the following step during forward selection:				
	Selected by Step 1	Selected by Step 2	Selected by Step 3	Selected by Step 4	Selected by Step 5
Number of Outpatient encounters ^a^	68.0%	69.6%	69.6%	69.6%	70.4%
Number of Total encounters ^b^	23.2%	24.8%	24.8%	24.8%	25.6%
Output Blood Pressure Taken ^c^	6.4%	40.0%	44.0%	46.4%	55.6%
ICD Code: Pervasive and specific developmental disorders (ICD-10: F80-F89)	1.6%	11.2%	12.0%	14.4%	17.6%
ICD Code: General symptoms and signs (ICD-10: R50-R69)	0.8%	4.0%	6.4%	17.6%	23.2%
Most Recent BMI ^d^	0.0%	10.4%	13.6%	14.4%	16.0%
Total number of high-level medication groups	0.0%	9.6%	18.4%	21.6%	23.2%
Normal Vaccine Requirement	0.0%	8.0%	12.8%	19.2%	27.2%
ICD Code: Body Mass Index BMI (ICD-10: Z68)	0.0%	4.8%	34.4%	44.0%	50.4%
ICD Code: Mood [Affective] disorders (ICD-10: F30-F39)	0.0%	3.2%	6.4%	7.2%	7.2%

a. Includes specialty and primary care outpatient appointments

b. Includes all pediatric patient encounters (outpatient, urgent care, emergency department and hospital encounters) in the specified time frame

c. Yes/No variable of documentation of a blood pressure taken during an outpatient encounter

d. BMI recorded most recently to first CAST visit

**Fig 1:**
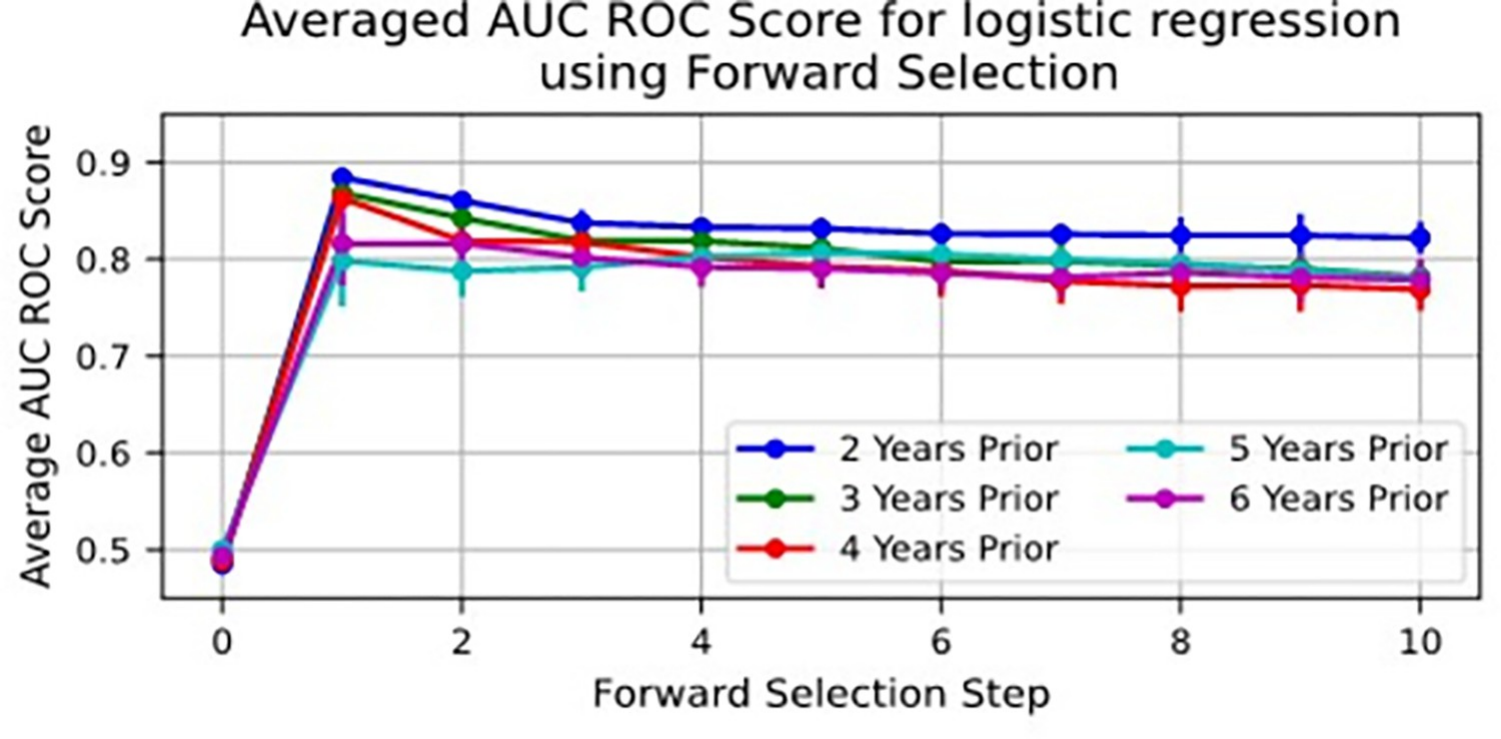


Fig 1 shows the averaged ROC-AUC Scores for the 25 models developed at each step of the forward selection process for each time window. For each time window, the feature selected at Step 1 of forward selection increased the AUC score from an initial 0.5 to between 0.81 and 0.88. In other words, the features “Number of Outpatient Encounters” and “Number of Total Encounters,” which were selected at Step 1 of forward selection in over 90% of the models, showed good ability to predict timely transfer to adult care. The highest AUC score, 0.88, was achieved at the 1^st^ forward selection step for the Year 2-time window. Steps after the first did not improve the model AUC score for 2-, 3-, and 4-years prior models. The 2-year prior timeframe had consistently higher scores than the other timeframes.

## Discussion

In this study, we found that total number of outpatient encounters in pediatric care and total encounters–highly correlated variables–were associated with timely transfer to adult care. Notably, this one characteristic explained most of the variability in the models we looked at, and no other patient characteristic was consistently included in the models.

This finding has multiple possible explanations. One possible explanation is that patients who attend many appointments (or their families) have developed effective strategies for scheduling and attending appointments. In this scenario, the fact that patients attend their appointments in pediatric care is a potential marker that they are equipped to make appointments in adult care and may not need as much support with transfer to adult care as patients who do not attend as many appointments. Another explanation is that transition education and support is currently occurring in the context of patient appointments [19]. Thus, patients who attend appointments are the ones getting the crucial education and support needed to make the transition to adult health care. Regardless of the explanation, patients who do not attend as many appointments in pediatric care are less likely to make a timely transfer to adult care, either because they have difficulty attending appointments in general or because they did not receive education and support to assist with the transfer, or they experience some other unknown barrier to timely transfer.

Clinically, this finding has some immediate applicability to practitioners providing care to autistic adolescents and young adults who are due for transfer to adult care. Practitioners have reported that a lack of time and resources are barriers to transition [20, 21]. Our data suggest that patients attending fewer appointments are more likely to need extra support transferring to adult health care in a timely fashion. So, if a clinic is trying to determine who may need help with transition and transfer, the answer may simply be to ask, “Who haven’t we seen in a while?” Until health systems are better equipped to support adolescents and young adults, including those on the autism spectrum, during their transition, these kinds of quick assessments can be of great value to practitioners as they seek to support their patients during their transition to adult health care with limited resources. Some of the responses that practices could consider when they notice a patient hasn’t attended an appointment in a while include sending general information about transition to families via mail, email, or the electronic health portal, so that even families who attend fewer appointments are sent important information about the transition and transfer to adult care. Practices could also consider scheduling additional appointments to plan specifically for transferring to adult care.

It is important to consider that an additional possible explanation of our findings is that more complex patients are more likely to make a timely transfer to adult care. In this scenario, the total number of appointments is primarily a reflection of a need for lots of care. We did not specifically look at the show rate in this study, and so the possibility that patients who need a lot of care make the transfer successfully should be considered. Families of patients with complex chronic conditions, including autism, have noted a number of barriers to transferring to adult care [2, 3, 22]. Given the barriers that families have encountered, one might expect complexity to be a marker of difficulty transferring to adult health care. Perhaps for patients with many medical needs and their families, significant priority is given to making the transfer to adult care. Perhaps those who are complex get more support than the less complex. Future work is needed to determine if our findings here are reflective of a high show rate to appointments generally or are a marker of patient complexity.

From a research perspective, replication studies in other institutions and in other patient populations are needed. We were specifically evaluating the transfer between one pediatric hospital and one adult clinic, which focused on providing primary care to autistic adults. The experience of those who did not have a set clinic or clinics to transfer to may be quite different, as may those with conditions such as type I diabetes or inflammatory bowel disease, which have less impact on development than autism. Replication studies would benefit from including larger study populations than ours. High levels of collinearity among certain variables and a relatively small study population made it difficult to assess independent contributions from each variable and limited the analytic strategies that could be applied. We accounted for this difficulty with forward selection, but larger studies would be an even better way to address the collinearity concern.

Another important limitation of this paper is that we only studied those patients that eventually participated in the CAST program, regardless of whether their transfer met our definition of being timely. Patients who were referred to CAST by an NCH practitioner or clinic but never attended are not included. The patients who never established with CAST may represent a different population, and we have not accounted for them in this study.

Finally, we feel that it is important to acknowledge that the population studied here was mostly white and mostly male. The proportion of white patients in this study is similar to the proportion of white patients in Ohio, and so is likely representative of this area. Similarly, the proportion of male patients is similar to other studies of autism. However, the limitations remain.

## Conclusion

Despite the limitations, we feel this paper represents an important step in improving our understanding of the transfer from pediatric to adult care for autistic adolescents and young adults. The work showed quite emphatically that completing many appointments prior to the transfer to adult care is correlated with a timely transfer to adult care. More work is needed to understand why this relationship exists. In the meantime, practitioners can consider reaching out to patients who haven’t had many appointments in recent years to assess the need for transition support for those patients and families, since the patients and families with many appointments generally make the transfer to adult care in a timely fashion.

## Abbreviations

CAST: Center for Autism Services and Transition
NCH: Nationwide Children’s Hospital
EMR: electronic medical record
CMS: Centers for Medicare & Medicaid Services
ICD-10: International Classification of Diseases, Tenth Revision
HPV: Human Papilloma Virus
BMI: body mass index
AIC: Akaike Information Criterion
ROC: receiver-operator curve
AUC: area under the receiver-operator curve
